# Case Report: Endocapillary Glomerulopathy Associated With Large Granular T Lymphocyte Leukemia

**DOI:** 10.3389/fimmu.2021.810223

**Published:** 2022-01-25

**Authors:** Tao Zhao, Nan Hu, Xiaojuan Yu, Tao Su

**Affiliations:** Department of Nephrology, Peking University First Hospital, Beijing, China

**Keywords:** large granular lymphocyte (LGL) leukemia, acute kidney injury, endocapillary glomerulonephritis, histology, immunohistochemistry

## Abstract

Large granular T lymphocyte leukemia (T-LGLL) is a rare indolent lymphocyte leukemia. The clonal proliferation of T cells, which is related to STAT3 gene mutation and abnormal Fas-mediated apoptosis pathway after cell activation, plays a major role in disease progression. Some studies have found that the exogenous and continuous stimulation of endogenous antigens, such as virus infection, is related to the pathogenesis of T-LGLL. The renal pathological manifestations of T-LGLL have rarely been described. In this study, we report a case of T-LGLL with kidney involvement as proteinuria, acute kidney injury, with the appearance of circulating T-LGL infiltrating intra-glomerular capillaries, and endocapillary glomerulopathy. We also summarize reported cases of renal injury associated with LGLL.

## Introduction

Large granular T lymphocyte leukemia (T-LGLL) is a rare type of lymphoproliferative disorder characterized by the clonal expansion of large granular T lymphocytes (LGLs) (1, 2). The abnormal proliferation of LGLs is usually associated with hematologic disorders and autoimmune diseases. Kidney disease can be rarely recognized in T-LGLL patients. Here we described a case of acute kidney injury associated with T-LGLL. A diagnosis of endocapillary glomerulopathy was confirmed by histology analysis, revealing the endocapillary infiltration of LGLs.

A 63-year-old man was admitted to our Department of Nephrology for further investigation of massive proteinuria. He had a 2-year history of rheumatoid arthritis (RA) and splenomegaly. At 1 month before admission, the patient was diagnosed with T-LGLL accidentally when he was experiencing an exacerbation of upper respiratory tract infection (fever of 38.2°C with cough and sputum), with a lymphocyte count of 5.68 × 10^9^/L showing the presence of specifically large lymphocytes containing granules and a flow cytometry analysis showing a CD2+/CD3+/CD4−/CD7+/CD8+/CD57+/TCRαβ+ phenotype. The bone marrow TCR gene analysis showed a rearrangement of TCRB and TCRG gene clones. At that time, the serum creatinine (SCr) was 90 μmol/L, whereas he developed progressive edema and proteinuria 10 days later.

On admission, the patient complained of anorexia. Upon physical examination, it was found that the blood pressure was at 180/100 mmHg. There was severe edema, no rash, no palpable superficial lymph nodes, and no sign of livedo reticularis indicating cryoglobulinemia. The laboratory examination revealed acute kidney injury in that SCr was rapidly elevated to 327 μmol/L, with blood urea nitrogen (BUN) at 63.69 mmol/L, BUN to SCr ratio ≈60:1, uric acid at 714 μmol/L, and albumin at 30.4 g/L (40–55). The complete blood count results were as follows: WBC, 5.4 × 10^9^/L; Hb, 96 g/L; reticulocyte, 176.3 × 10^9^/L (24–84); platelet, 76 × 10^9^/L; neutrophil, 10.7%, 0.6 × 10^9^/L (1.8–6.3); and lymphocyte, 84.8%, 6.8 × 10^9^/L (1.1–3.2). The urinalysis found microscopic hematuria at 4–7 cells/HPF, the 24-h urinary protein was at 10.85 g, the 24-h urinary sodium was at 12.75 mmol, and the filtered sodium excretion fraction was at 0.2%. The tests for antinuclear, antineutrophil cytoplasmic, anti-GBM antibodies, hypocomplements (C3 and C4), and cryoglobulin did not detect their presence; the anti-cyclic citrulline polypeptide antibody was at 140 RU/ml (<5), and antistreptolysin O (ASO) was 235 IU/ml (<220). However, the systemic inflammatory response was mild (erythrocyte sedimentation rate, 28 mm/h; hypersensitive C-reactive protein, 13.2 mg/L). The CT scan showed bilateral pleural effusion, pericardial effusion, enlarged kidneys, and spleen as well as a large amount of peritoneal effusion.

**Figure 1:**
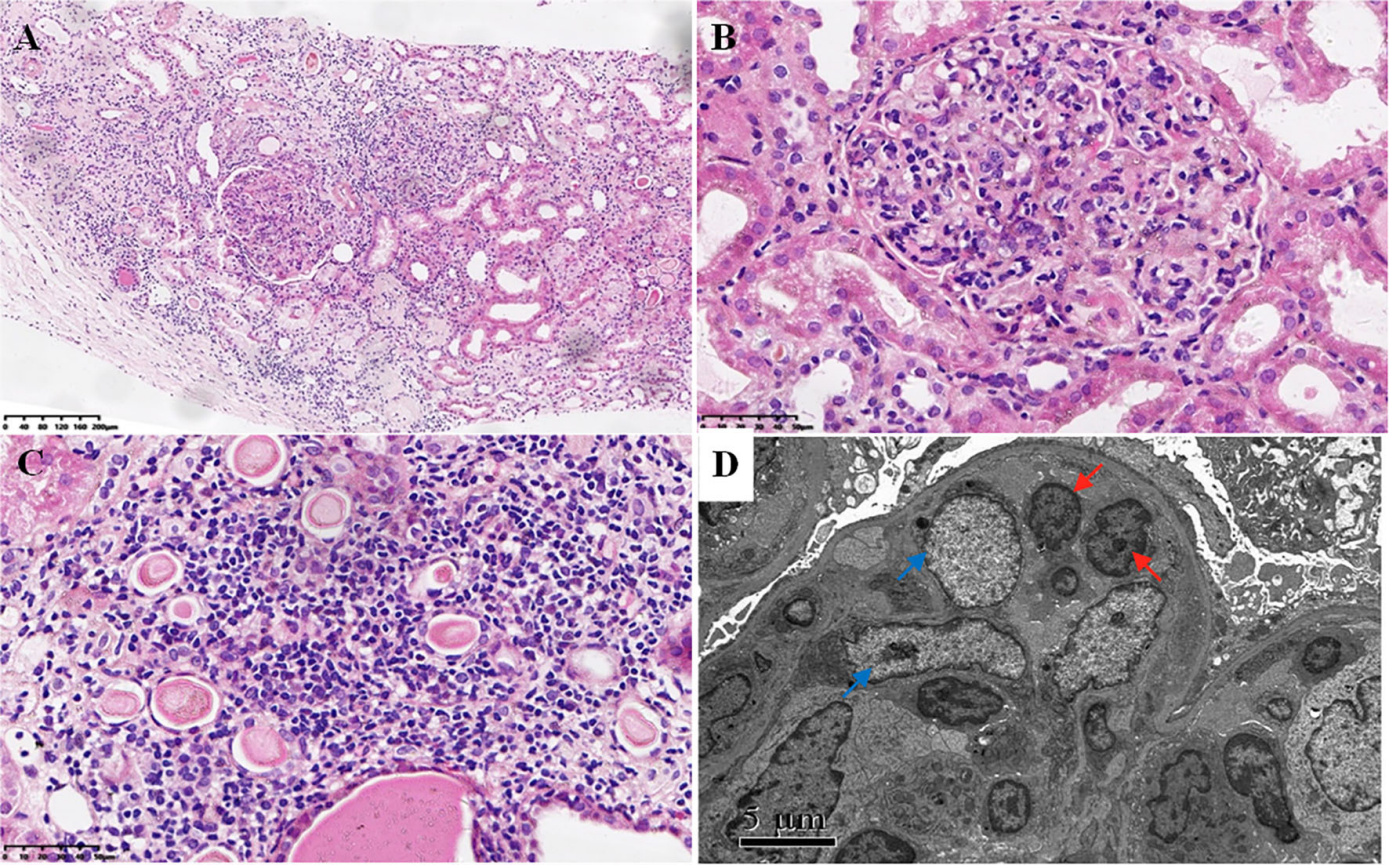
Light and electron microscopy analysis. **(A–C)** Light microscopy using hematoxylin–eosin staining showing diffuse endocapillary proliferation composed of heteromorphic lymphocytes and a small number of neutrophils **(A, B)** and multifocal interstitial infiltration of lymphocytes and monocytes with focal tubular necrosis **(C)** [magnification, ×100 **(A)**; magnification, ×400 **(B, C)**]. **(D)** Electron microscopy showing endocapillary proliferation of heteromorphic lymphocytes (red arrows) and low-density electron-dense deposition in the mesangial area (blue arrows) with the extensive fusion of podocyte foot processes.

**Figure 2:**
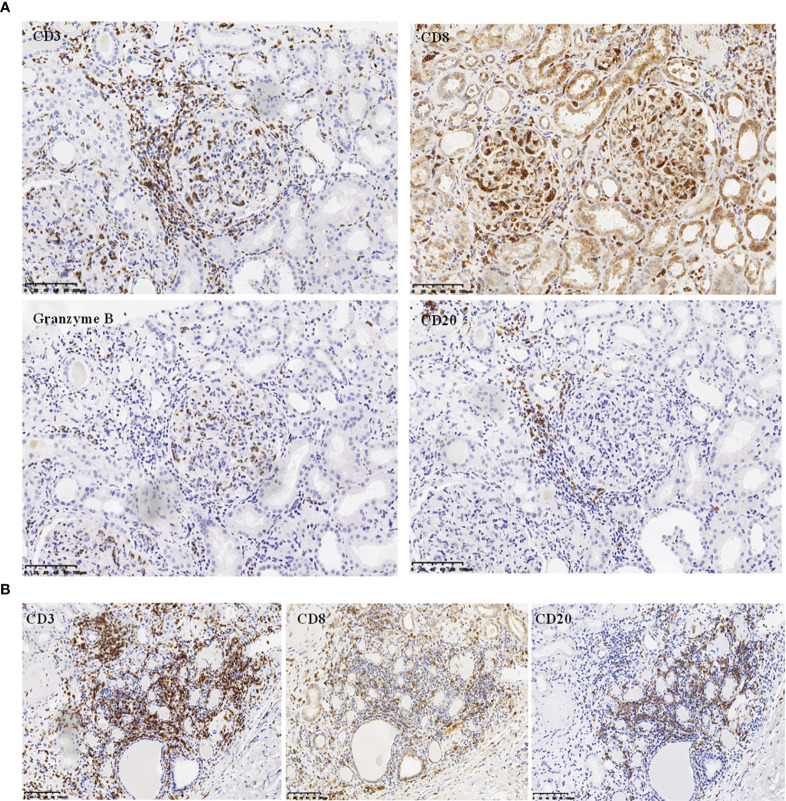
Immunohistochemistry analysis. **(A)** Immunochemistry analysis showing CD20^-^/CD3^+^/CD8^+^/GranzymeB^+^ phenotype of the infiltrating endocapillary glomerular cells (magnification, ×200). **(B)** Immunochemistry analysis on tubulointerstitial compartment showing CD20^+^/CD3^+^/CD8^+^ cells infiltrating the interstitium (magnification, ×200).

The renal pathological findings showed that the glomeruli were negative for IgG, IgA, C1q, FRA, ALB, IgG1, IgG2, IgG3, and IgG4, with IgM+ and C3+++ being positive and granularly depositing in the mesangial area by immunofluorescence analysis. Under light microscope observation, there were 21 global ischemic sclerosis of a total of 59 glomeruli, which were mainly distributed beneath the renal capsule. The non-sclerotic glomeruli had an appearance of diffuse endocapillary proliferation. Most heteromorphic lymphocytes and a small number of neutrophils were observed in the intra-glomerular capillaries (**Figure 1**). No structure of glomerular crescent and arteriolitis were detected. There was focal tubular necrosis, multifocal lymphocytes, and mononuclear cells inundates with interstitial fibrosis; however, tubulitis was not found. Under electron microscope observation, segmental low-density electron-dense deposition was disclosed limitedly in the mesangial area with the extensive fusion of podocyte foot processes, and no crystal or special microstructure was observed. Furthermore, the immunohistochemistry staining verified that the infiltrated heterotypic lymphocyte intra-glomerular capillaries expressed CD20-/CD3+/CD4-/CD8+/TCRβ-/TIA1+/GranzymeB+/CD2-/CD7+/CD56-/Ki67 5%, demonstrating a cytotoxic T cell immunophenotype (**Figure 2**). The above-mentioned findings of renal pathology suggested endocapillary proliferative glomerulonephritis associated with T-LGLL. Moreover, considering the history of recent respiratory infection, increased ASO, and C3 deposition in the mesangial area, infection-related glomerulonephritis could not be excluded. The extremely decreased FeNa indicated intra-glomerular hypoperfusion, which could contribute to aggravated tubular injury and kidney function loss. In addition, lymphocyte and monocyte (**Figure 1C** and **Figure 2**) interstitial infiltration was revealed in histology, proving that tubulointerstitial injury might also be involved in the pathogenesis of the kidney injury.

**Figure 3:**
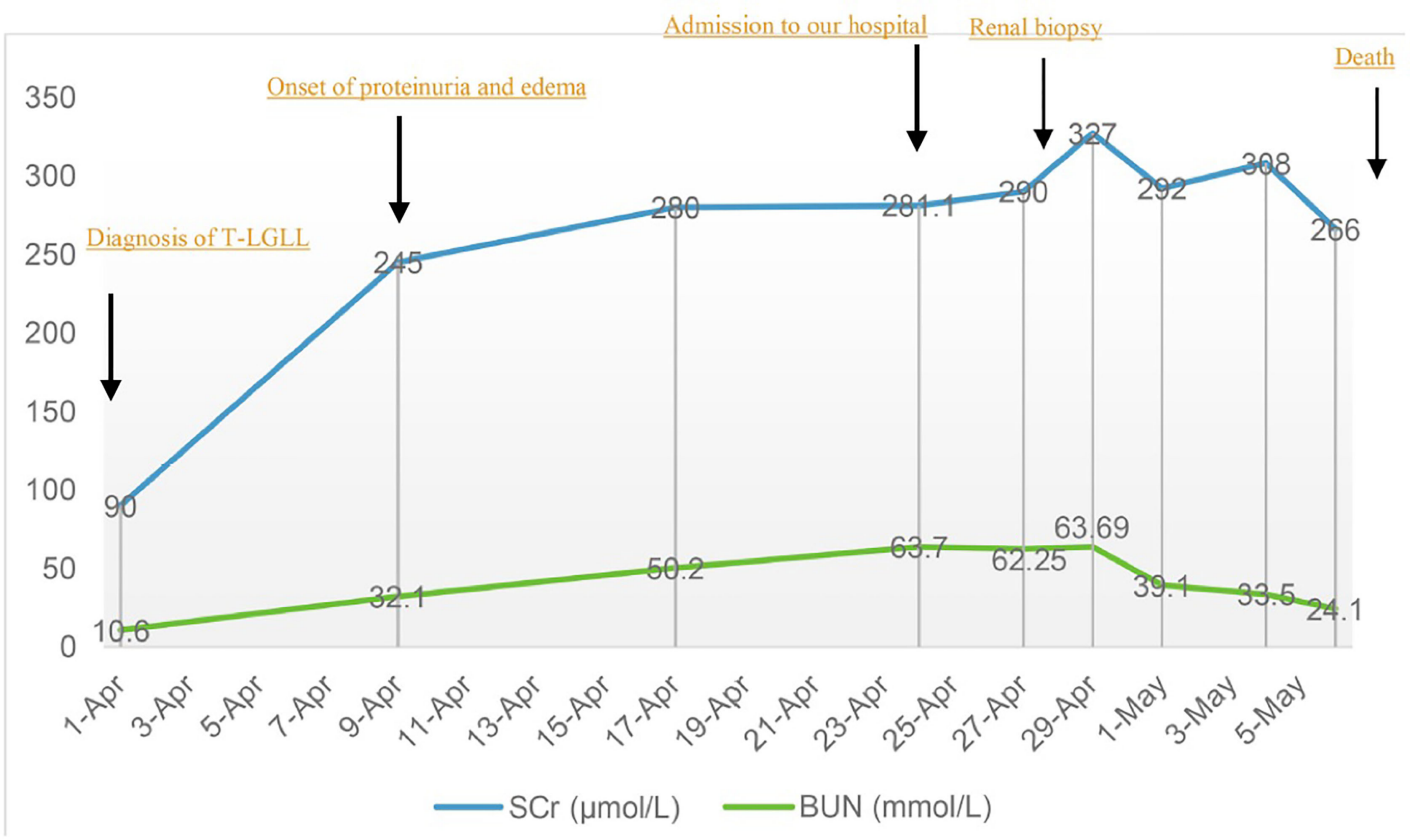
Changes of serum creatinine and blood urea nitrogen during the disease course.

Supportive therapy was initiated after the patient was admitted to our hospital, including hemodialysis and transfusion of platelet and plasma, while the levels of SCr and BUN continued to increase as shown in **Figure 3**. The peripheral levels of platelet (68–93 × 10^9^/L) and neutrophil (0.4–1.4 × 10^9^/L) remained low. The coagulation function test revealed prolonged activated partial thromboplastin time (35–40 s) and decreased fibrinogen (1.65–2 g/L), strongly suggesting the occurrence of disseminated intravascular coagulation. Immunosuppressive therapy was denied by the patient after the final diagnosis was confirmed. He chose to transfer to a local hospital where supportive treatment was sustained and unfortunately died a few days later.

## Discussion

T-LGLL is a common type of LGL leukemia. However, the renal involvement of T-LGLL is rare. In the present study, we reported a case of T-LGLL-induced endocapillary proliferation. The current diagnosis of T-LGLL is mainly based on clinical manifestations, cell morphology, immunophenotype, and T cell clonality (3, 4). The typical clonal proliferating T lymphocytes (T-LGL) are mostly killer effector cytotoxic T lymphocytes, leading to tissue damage. Their abnormal proliferation causes an imbalance of lymphocyte subsets and secretes inflammatory cytokines (such as FasL and IL-18) (5, 6), leading to renal injury. Ribes D et al. (5) reported that the supernatant of cultured peripheral blood mononuclear cells, including clonal T cells, triggered the phenotype switch of the HK-2 cell line from a quiescent to a pro-inflammatory state, characterized by a pro-inflammatory state by NF-κB nuclear translocation and overexpression of inflammatory cytokines or chemokines. This suggests that circulating T-LGL could directly activate tubular epithelial cells or stimulate the immunoreactivity of T/B cell populations, which could contribute to kidney injury. Orman et al. (7) reported a case of T-LGLL-associated nephrotic syndrome, of which the clinical behavior favored minimal-change glomerulopathy (though a kidney biopsy was not undertaken), suggesting that cytotoxic T-LGL could disrupt the normal anionic charge of glomerular capillary walls independent of heavy local infiltration. Audemard et al. reported three among 10 cases of T-LGLL with cryoglobulin-associated vasculitis, renal insufficiency, and membranoproliferative glomerulonephritis without renal infiltration of T-LGL (8). They all had systemic involvement, including purpura, polyneuritis, and arthritis. Abnormal B-cell activities in LGLL patients could also induce kidney impairment. Zhang M et al. reported crescentic glomerulonephritis induced by anti-GBM disease in a patient of T-LGLL (9). There is also a case report of AH renal amyloidosis type (γ1) with T-LGLL (10). More recently, Pierre I et al. reported a case of natural killer cell LGL-induced glomerulonephritis, which showed global endocapillary proliferation with a marked predominance of circulating and interstitial infiltrating NK cells, IgM, C3 deposit, tubulitis, and tubular necrosis (11). Our current patient had similar pathological features of endocapillary proliferative glomerular changes, which originated from a cytotoxic T lymphocyte subset. These activated lymphocytes express cytotoxic granule protein (TIA-1 and GranzymeB) and had strong cytotoxic effects. Previous genetic testing found that IL1B gene was silenced in CD4^-^/CD8^+^ T cells of normal individuals but activated in LGL-T cells (5). IL-1β, as an effective proinflammatory cytokine, is known to be associated with tumor necrosis factor α, playing a central role in tissue injury of RA accompanied by T-LGL leukemia. Interestingly, the glomerular infiltrating clonal T cells of the patient in this report showed an immunophenotype consistent with T-LGL, but with low aggressiveness, and his renal biopsy revealed polymorphic inflammatory cell interstitial infiltrates. Even so, interstitial inflammation also remained one of the factors for acute kidney injury (AKI). The clinical and pathological characteristics of similar cases reported in the literature are summarized in **Tables 1** and **2**.

**Table 1:** Clinical features of reported cases of renal injury associated with large granular lymphocyte leukemia.

Patient	Age/sex	Subtype LGLs	Peripheral LGL^a^	Hematological abnormalities	Symptoms	Autoimmune disorders
1	57/F	T-LGL	73%	L, N,	Fatigue, nausea, choking, neuropathic symptoms	M-protein shown in SPEP and UPEP
2	78/M	NK-LGL	72%	L	NS	NS
3	74/F	T-LGL	0.45 × 10^9^/L	Hb	Purpura, polyneuritis	Cyroglobulinemia mixed type 2 with IgM
4	59/F	T-LGL	0.63 × 10^9^/L	Within normal range	Purpura, arthritis	Cyroglobulinemia
5	69/F	T-LGL	0.45 × 10^9^/L	N,	Purpura, arthritis	Cyroglobulinemia
6	37/M	T-LGL	16% (0.32 × 10^9^/L)	NS	NS	Anti-GBM disease
7	51/M	T-LGL	75%	L, PLT	“B” symptoms, hepatosplenomegaly	NS
8	63/M	T-LGL	72.7%	L, N, Hb, PLT	Splenomegaly, fever	Rheumatoid arthritis

L, lymphocyotosis (lymphocyte >4 × 10^9^/L); N, neutropenia (neutrophil <1.5 × 10^9^/L); Hb, anemia (hemoglobin >11 g/dL); PLT, thrombocytopenia (platelet <150 × 10^9^/L); NS, not stated.

a Presented as ratio or absolute count.

8 is our currently reported case.

**Table 2:** Characteristic of renal disease in reported cases of renal injury associated with large granular lymphocyte leukemia.

Patient	Time of diagnosis of renal injury compared to LGL	Clinical course	SCr (µmol/L)	Proteinuria	Renal histology
1	14 years after	Progressive proteinuria (0.5–1.5 g/h in 17 months)	74.26	UTP: 2.5 g/24 h	AH amyloid (mostly restricted to glomeruli), moderate chronic changes (including glomerulosclerosis)
2	3 years after	AKI	220	ACR: 2 g/g	Endocapillary glomerulonephritis and tubulitis with LGL NK cell infiltration seen in both areas
3	Synchronous	NS	NS	NS	MPGN without LGL infiltration
4	6 years before	NS	NS	NS	NS
5	2 years before	NS	NS	NS	Endocapillary glomerulonephritis
6	1 year before	AKI	295	UTP: 6.92 g/24 h	Crescentic glomerulonephritis and segmental membranous nephropathy, with strong linear GBM staining on direct immunofluorescence
7	Synchronous	Nephrotic syndrome	132.6	UTP: 8.9 g/24 h	Presumptive minimal-change nephropathy suggested by clinical behavior
8	1 month after	AKI	327	UTP: 10.85 g/24 h	Endocapillary glomerulonephritis with heterotypic lymphocyte infiltration, probable post-infection glomerulonephritis

NS, Not Stated.

However, the exact risk factors attributing to AKI in this T-LGLL patient were complicated. As we know, secondary neutrophil deficiency is a disadvantageous factor of innate immunity, which makes patients susceptible to infection. Infection-related nephritis would also be considered if patients presented with nephritis syndrome. We noticed that the patient had an extremely elevated BUN-to-SCr ratio and decreased FeNa. The renal biopsy revealed ischemic glomerulosclerosis, involving 35.6% of the glomeruli. We speculated that the intra-glomerular hypoperfusion was derived from severe endocapillary proliferative changes. All these could lead to peri-tubular capillary ischemia and tubular injury.

Our patient has a 2-year history of splenomegaly and RA with a positive anti-CCP antibody. Splenic involvement is almost universal in LGLL patients, manifested as splenomegaly and thrombocytopenia, which is closely related to autoimmune diseases. RA is most commonly described in 11–36% of patients, also including Felty syndrome, scleroderma, polymyositis, various types of vasculitis, Sjogren’s syndrome, and Behcet’s disease (12). The clonal T-LGL was reported to be detected in 3.6% of RA. The onset of LGLL is indolent; thus, diagnosis and specific treatment are easily delayed. Experts recommend the initiation of a specific treatment for T-LGLL with relevant autoimmune diseases (13). However, our patient presented rapid deterioration of kidney function with poor prognosis. One explanation was that the T-LGLL might have occurred a few years prior to the diagnosis of splenomegaly and RA. Insufficient attention to the history of splenomegaly might result in delayed diagnosis of T-LGLL and absence of timely treatment, consequently leading to disease progression.

Our report contained several limitations. Firstly, the reasons for the death of the patient need more analysis. However, clinical information concerning follow-up in the local hospital was lacking. Thus, it was hard to find out the exact cause of his death. Secondly, to further evaluate the pathogenesis of peripheral T-LGL, it is necessary to measure the serum inflammatory cytokines produced by the activated cytotoxic T cells, such as FasL and IL-18. Unfortunately, the patient died within a few days after the diagnosis, and we have not obtained consent from him.

### Concluding Remarks

In conclusion, this case is a rare report of T-LGLL-induced AKI presenting endocapillary proliferative glomerulonephritis. We aim to arouse more attention to the kidney presentation of this clonal T cell proliferative disease. The significance lies in the fact that early recognition of T-LGLL-associated organ-specific injury and timely initiation of therapy would be beneficial to improve the disease outcome.

## Data Availability Statement

The original contributions presented in the study are included in the article/supplementary material. Further inquiries can be directed to the corresponding author.

## Ethics Statement

Written informed consent was obtained from the individual(s) for the publication of any potentially identifiable images or data included in this article.

## Author Contributions

NH collected data and reviewed the literature. TZ and NH drafted the manuscript. The diagnosis was confirmed clinically by TS and NH. XY made the pathological diagnosis and generated **Figures 1** and **2**. TS, NH, and TZ reviewed and edited the manuscript. All authors contributed to the article and approved the submitted version.

## Funding

This research was supported by the National Science and Technology Major Projects for major new drugs innovation and development (Grant 2017ZX09304028 to TS), and Chinese Academy of Medical Sciences Innovation Fund for Medical Sciences (No. 2019-I2M-5-046).

## Conflict of Interest

The authors declare that the research was conducted in the absence of any commercial or financial relationships that could be construed as a potential conflict of interest.

## Publisher’s Note

All claims expressed in this article are solely those of the authors and do not necessarily represent those of their affiliated organizations, or those of the publisher, the editors and the reviewers. Any product that may be evaluated in this article, or claim that may be made by its manufacturer, is not guaranteed or endorsed by the publisher.
